# Lessons Learned from the Design, Construction, and Commissioning of a Retrofitted Arthropod Containment Level 3 Insectary

**DOI:** 10.4269/ajtmh.22-0790

**Published:** 2023-05-15

**Authors:** Erin Lauer, Kelly N. Kim, Robert A. Dettmann, Adam E. J. Fleming, Gregory L. Powell, Amanda D. Rice, Giorgio Scarpellini, Rocco Casagrande, David R. Gillum

**Affiliations:** ^1^ Gryphon Scientific, LLC, Takoma Park, Maryland;; ^2^ Environmental Health and Safety, Arizona State University, Tempe, Arizona

## Abstract

Arthropods are vectors for many pathogens that significantly harm human and animal health globally, and research into vector-borne diseases is of critical public health importance. Arthropods present unique risks for containment, and therefore insectary facilities are essential to the safe handling of arthropod-borne hazards. In 2018, the School of Life Sciences at Arizona State University (ASU) began the process to build a level 3 arthropod containment (ACL-3) facility. Even with the COVID-19 pandemic, it took more than 4 years for the insectary to be granted a Certificate of Occupancy. At the request of the ASU Environmental Health and Safety team, Gryphon Scientific, an independent team with biosafety and biological research expertise, studied the project lifecycle through the design, construction, and commissioning of the ACL-3 facility with the goal of identifying lessons learned from the delayed timeline. These lessons learned convey insight into best practices for assessing potential facility sites, anticipating challenges with retrofitted construction, preparing for commissioning, equipping the project team with necessary expertise and expectations, and supplementing the gaps in available containment guidance. Several unique mitigations designed by the ASU team to address research risks not specifically addressed in the American Committee of Medical Entomology Arthropod Containment Guidelines are also described. Completion of the ACL-3 insectary at ASU was delayed, but the team thoroughly assessed potential risks and enabled appropriate practices for the safe handling of arthropod vectors. These efforts will enhance future ACL-3 construction by helping to avoid similar setbacks and streamlining progress from concept to operation.

## INTRODUCTION

Arthropods are vectors for a myriad of bacterial, viral, and parasitic infections that threaten human and animal health globally. Approximately 725,000 deaths worldwide each year are attributed to mosquito-borne diseases alone.^1^ Research into understanding the interplay between the disease agents and vectors is critical to support public health and medicine.

Arthropod research is associated with unique biosafety risks. Their small size, rapid motility (i.e., flying, jumping, climbing), and diverse life cycle stages pose opportunities for evading detection and escape. Accidental escape of arthropods could lead to the establishment of a nonnative species in the local environment, and, potentially, to the risk for the eventual introduction and changes in the pattern of transmission of vector-borne diseases. Infected arthropods, on the other hand, present an immediate risk of infection with vector-borne disease to local human and animal populations, with the possibility of the establishment of exotic diseases in a new area. The potential for severe consequences of escape warrants stringent biosafety measures for handling arthropods, and thus specialized insectary facilities are essential to safe infectious disease research with these disease vectors.

Responsible conduct of arthropod research in the United States is guided by several government entities in accordance with set standards for biocontainment. These standards are espoused in various government advisory documents—including the National Institutes of Health (NIH) Design Requirements Manual,^2^ NIH Laboratory Sealant Requirements,^3^ Animal and Plant Health Inspection Service (APHIS) Guidelines for Containment of Nonindigenous Phytophagous Arthropods and Their Parasitoids and Predators,^4^ and the Biosafety in Microbiological and Biomedical Laboratories manual^5^—as well as additional inspection checklists available upon request from the CDC^6^^,^^7^ and the United States Department of Agriculture (USDA).^8^ Published by the American Committee of Medical Entomology, a subcommittee of the American Society of Tropical Medicine and Hygiene, the Arthropod Containment Guidelines provide targeted guidance for the diverse but unique nature of handling arthropods.^9^ Together, these materials form a disjointed collection intended to inform the assessment of risk, outfitting of laboratories, and establishment of safe research practices in an insectary laboratory.

**Figure 1.**
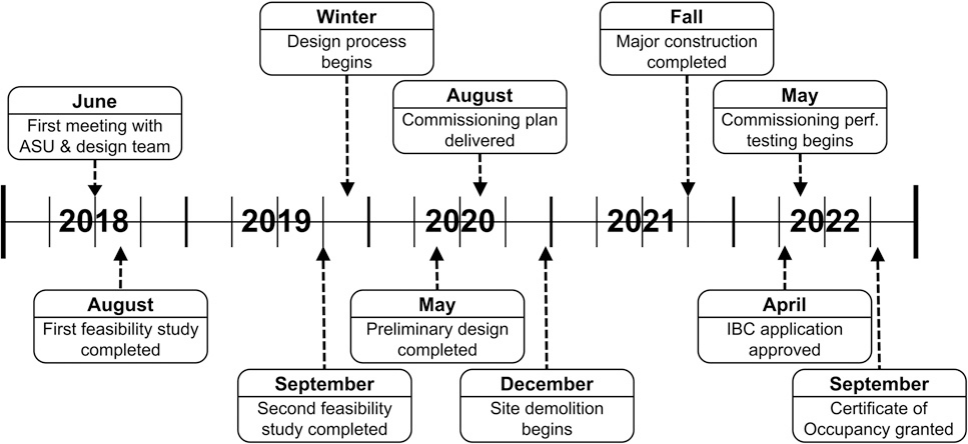
Timeline of the Arizona State University Insectary Project.

In 2018, the School of Life Sciences at Arizona State University (ASU) recognized the need for an insectary to support their expanding research mission and began the process to build a level 3 arthropod containment (ACL-3) facility. Four years later, the insectary had reached the commissioning stage after a series of setbacks and delays (Figure 1). The ASU Environmental Health and Safety team (EHS) is committed to responsible conduct of research and played a critical role in the planning, design, and execution of the facility construction to ensure proper containment standards. The team leveraged their decades of combined experience in biosafety, their professional networks, and all available biosafety guidance to direct the process. Additionally, the broader project team comprised a wide range of personnel with experience in research laboratories respective to their roles in architecture and design, engineering, construction, project management, and commissioning.

Although equipped with broad and deep expertise, completion of an insectary at ASU was confronted by a series of challenges that ultimately delayed research of public health importance. As a result, this independent study was initiated to examine the insectary project—through the planning, design, construction, and commissioning of the facility—for retrospective insight into the obstacles encountered and their root causes. These lessons learned could be used by the broader biosafety community in the future to inform the delivery of new containment facilities while still upholding the highest commitment to safety.

## MATERIALS AND METHODS

The EHS team at ASU invited Gryphon Scientific to conduct an independent review of the process to design and build the ACL-3 facility with the goal of identifying lessons learned from the delayed timeline. Gryphon Scientific is a life sciences research and consulting company with experience investigating and analyzing best practices in biosafety around the world.

Our team first reviewed all biosafety standards and guidance that were used by the insectary project team to navigate the design and construction process and ensure the facility would pass all necessary inspections and commissioning. These materials include guidelines published specifically for work with arthropod vectors and for general biosafety and laboratory design as well as regulatory inspection checklists for ACL-2 and ACL-3 laboratories that were procured directly from the respective agencies, all of which are listed in Table 1.

**Table 1:** Documentation used to guide arthropod containment requirements

Type of guidance	Document title	Publisher
Arthropod-specific	Arthropod Containment Guidelines, Version 3.2^9^	ACME, ASTMH
	Containment Guidelines For Nonindigenous, Phytophagous Arthropods and Their Parasitoids and Predators^4^	APHIS, USDA
Laboratory biosafety	Biosafety in Microbiological and Biomedical Laboratories (BMBL)^5^	CDC and NIH, HHS
	Design Requirements Manual (DRM)^2^	NIH
	NIH Laboratory Sealant Requirements^3^	NIH
Inspection checklists	USDA Inspection Checklist for ACL-3 Laboratories^8^	USDA
	CDC Import Permit Inspection Checklist for Arthropod Containment Level 2 and Containment Level 3^6^^,^^7^	CDC

After completing background research, the Gryphon team visited the ASU campus to tour the in-progress facility and convene with the EHS team onsite. The EHS personnel involved with the insectary project are experienced with high-containment programs, animal biosafety, laboratory management, environmental safety, occupational health, and biosecurity. The Gryphon team developed a programmatic guide to ensure that oral data were collected consistently from all parties. Gryphon interviewed each member of the EHS team individually to extract their professional experience and candid perspective on the process. Questions focused on not only the difficulties of planning and constructing a high containment space but also on where those difficulties arose unexpectedly. Despite the project team’s extensive experience and available guidance, the intent was to reveal resource gaps. Group discussions were also facilitated by the Gryphon team to gather additional context and background on the project.

Following this visit, the Gryphon team conducted interviews with representatives from other critical parts of the insectary project team beyond EHS. Discussions were held with involved members of the planning, design, engineering, construction, project management, and commissioning teams to glean in-depth insight from all trades and perspectives. Records were kept from each conversation and then synthesized into overall trends and takeaways from across the entire project team that could bolster the efficiency and biosafety of future insectary laboratories.

## RESULTS AND DISCUSSION

This investigation gleaned several findings that have been distilled into lessons learned that should be considered in all future efforts to commission an arthropod research facility. We hope that these lessons from a delayed but overall successful project, summarized in Table 2, will act as a resource for other biosafety personnel and researchers interested in a facility that enables responsible and safe arthropod research.

**Table 2:** Summary of lessons learned

Project element	Lesson learned
Site selection	Assess current conditions and past inspection reports when determining site feasibility
	Consider additional labor costs associated with planning for a retrofitted space
	Incorporate unexpected setbacks into the financial projections and anticipated schedule for retrofitting
	Consider anticipated growth and future institutional research goals
Retrofitted construction	Consider that deficiencies from original site have to be renovated in addition to planned renovations to meet facility requirements
	Understand that new requirements in an existing space may lead to a mismatch between best practices and available options
	Consider integration of new equipment with preexisting technical systems and policies
Commissioning	Plan and perform functional testing throughout construction
	Use photo documentation throughout construction along with functional test results to demonstrate requirements are fulfilled
Project team	Balance the level of necessary communication with the need for collaboration amongst the project team
	Engage safety personnel early and continually
	Engage a commissioning agent with relevant experience in containment insectaries from the design phase forward
	Ensure that specialized experience with designing or constructing an insectary is included in the project team
	Set clear expectations on high level of requirements from project outset
	Manage expectations of scientific and other impacted personnel to mitigate any consequences of delays
Published guidance	Consolidate available guidelines into a set list of requirements specific to the individual facility
	Develop additional guidance or best practices to operationalize requirements in the guidelines
	Harness professional networks and open-source resources to fill in gaps in the guidance
Unique solutions	Stock the facility with extra spill kits to soak up potential flooding due to emergency shower use and the absence of a floor drain
	Install a mesh barrier inside the fume hood to ensure secondary containment in the presence of flying arthropods
	Devise custom kill tanks to ensure decontamination of noninfected liquids
	Institute a new protocol for decontamination of infected materials given unique risks

### Site selection.

As the first step in planning, ASU completed a formal assessment of potential sites for the ACL-3 laboratory. The feasibility study considered how the existing structure and mechanical systems of the sites would fulfill the requirements of the future insectary, as well as the cost and timeline for completion, and led to the selection of a previously constructed but never commissioned BSL-3 facility. The original performance test results and records of deficiencies for this space could not be located and therefore were not evaluated as part of the feasibility study. As a result, over the next several years, many of the setbacks in completing the insectary were due to unexpected issues that were left over and unaddressed from the original space. Although past inspection reports on the uncommissioned BSL-3 facility were unavailable at the time—which could be considered a separate issue on document archiving policies—this absence of information on the site should have been factored into the selection decision. Alternatively, certain functional testing could have been redone during the feasibility study or before construction began. This may have identified longstanding issues in the space, including the malfunctioning door locks and pass-through autoclave the team was later faced with. In addition to filling in gaps in information on the space, the site assessment should have verified the information they did have in the original design and system plans by evaluating the conditions of the space as it currently existed. This step would have revealed problems with the site, such as the aforementioned autoclave and door locks, but also would have shown that the site documentation was incomplete. Airflow design plans for the space did not show that supply air ducts were exhausting air into the ceiling plenum, which became another challenge for the team when this was uncovered during construction. Expanding the site assessment to include greater field observation of the space and either a review of prior inspection reports, if available, or retesting the performance of critical systems could have revealed problems with the site earlier and either better prepared the team for the forthcoming issues or led them to choose a better suited space.

Constructing a new facility would have circumvented these challenges entirely and, for obvious reasons, should be favored over retrofitting a site if financial resources and available space permit. When evaluating financial projections between new and retrofitted construction, consideration should be given to the additional costs that may arise during the process due to unexpected but required repairs to the site and associated costs in materials, time, and labor. Consideration should also be given to the additional cost of labor necessary for the team to assess the potential spaces, equipment, and systems when evaluating different sites to retrofit. In an effort to be financially responsible, the ASU project team initially planned to limit ceiling demolition and repurpose ductwork preexisting in the BSL-3 space. However, as construction proceeded, it became clear to the team that the original infrastructure was not going to meet the needs of the insectary, and so it was ultimately removed and replaced. The team learned that prioritizing immediate savings is not always the best long-term investment for either the schedule or cost. If funding or space is prohibitive of new construction, then teams should anticipate unplanned delays and costs.

When selecting a facility site, teams should consider not only the requirements of the current research program but also its growth and future needs. Although funding may restrict the maximum facility size, quickly outgrowing the space could lead to overcrowding in the laboratory, which presents biosafety risks itself, or the need to build another insectary entirely. Vector-borne diseases continue to be a global burden, and ASU’s commitment to advancing disease control is already resulting in expansion of the research program and personnel. This project demonstrated that planning for and constructing high-containment facilities that are time intensive should be viewed through the lens of future institutional research goals.

### Retrofitted construction.

Although retrofitting a facility may seem appealing due to the potential cost- and time-savings, it bears the burden of inheriting any problems with the space from original construction or deterioration over time. The project team was faced with a long list of these issues from the initial BSL-3 space because it was never commissioned for use. Pipes were leaking water, floors were unlevel, and the perimeter was not properly sealed, among other issues. In addition to the unaddressed issues of the space itself, retrofitting exposes the project to the challenges with the larger building that the original site is housed within. In this case, the team was presented with even more inherited challenges, including damaged phone lines and lack of hot water for the sinks and shower. Although solutions to these problems can be and were devised—whether through the addition of a new water heater to directly service the facility or through installation of ramps to level the floor—having to deal with inherited issues should be anticipated when retrofitting an insectary.

In addition to inherited deficiencies, existing equipment may also be inherited from the original space in an attempt to control costs. Even if this equipment is functioning properly, it may be lacking added safety features equipped on newer models. Once researchers had moved into the space, a hands-free sink kept from the previous laboratory caused a flood in the pathogen culture room when material inadvertently triggered the activation sensor. The sink did not have a flow timer that is commonly found on newer models, and the water overflowed the connected carboy and flooded the space until it was eventually deactivated by the laboratory manager. Although durable equipment can be safely reused, the team should fully evaluate any limitations with the protocols being used in the new laboratory. Ensuring experienced end users are involved in pressure testing these protocols is critical to avoid issues with commissioning or afterward when the space is in full use.

Redesigning a space for a different purpose than what it was originally intended for presents a mismatch between existing configurations and new requirements. Although the infrastructure may have suited the original laboratory, it was not ideally designed to handle the restrictions of a higher containment facility. The majority of the necessary configuration changes were accounted for during the design phase; however, others only became illuminated during construction. Engaging a commissioning agent earlier in the process may have helped to identify and remediate issues before they reached construction, which is further elaborated later in this analysis. In one case, the project team uncovered access panels to airflow control valves in the ceiling of what would become the flight room. Such penetrations in an area of freely flying arthropods presented an unacceptable level of risk for escape. The best practice would have been to position all mechanical access points in a bordering space outside of containment, such as a corridor, to enable easier and safer accessibility. Because reconfiguring the mechanical systems layout was cost prohibitive, the team decided to remove the access panels from the flight room to make the ceilings a continuous surface without penetrations. They marked the location of the control valves above, and when the time comes for either repair or maintenance, a full shutdown of the facility will occur to access the control values via excision of the ceiling drywall to gain access. The team conceded that addressing new risks within the confines of an existing space produces added challenges and suboptimal solutions.

Beyond the facility itself, consideration had to be given to integration within the entire building and institution. In preparation for commissioning of the insectary, the project team began testing some of the new equipment to find that the key card access system did not integrate with the main building access controls and the security system on the mosquito rearing chambers did not comply with university IT policy. Although the equipment fit the needs of the facility itself, this project showed that retrofitting needs to consider all aspects of technical infrastructure as well as the physical infrastructure and should be a consideration for engineering team planning and scope determination. Integrating the technical systems of the containment facility within the broader technical systems and policies requires early consideration and planning to avoid delays.

### Commissioning.

Because retrofitting presents additional challenges, early and continuous evaluation of progress toward successful commissioning of the facility is key. Functional testing should take place as early as possible to avoid the identification of problems late in the process. Maintaining directional airflow between the various rooms and doors within the facility proved to be a challenge for the project team; however, early testing and balancing allowed the team to resolve issues without causing any major delays. Other minor issues with the mechanical, electrical, plumbing, and access control systems were also identified in a first pass of functional testing but were resolved while other work continued.

On the other hand, testing emergency backup power for the space resulted in considerable delays. Because of the effect a full power shutdown would have on other equipment in the building, testing had to be conducted in conjunction with the annual building shutdown scheduled at the end of the academic year in May. Although the majority of the construction and installation was complete, the team had to wait months to proceed to this commissioning requirement. In May, the transition to back-up power generators uncovered that the pressure monitors that were being reused from the previous construction were not connected to emergency power. Further investigation of this issue revealed that the uninterruptible power supply device had to be upgraded to support the additional circuitry should an actual power outage occur. Additionally, restoring normal power to the facility damaged the electronic components of the autoclave, which necessitated replacement of its motherboard. Not only were there delays in scheduling the tests, but necessary subsequent repairs caused additional setbacks. Sufficient transition to emergency power is a requirement for commissioning, and this project showed that planning out functional testing well in advance of commissioning can help avoid delays in fulfilling such requirements.

**Figure 2.**
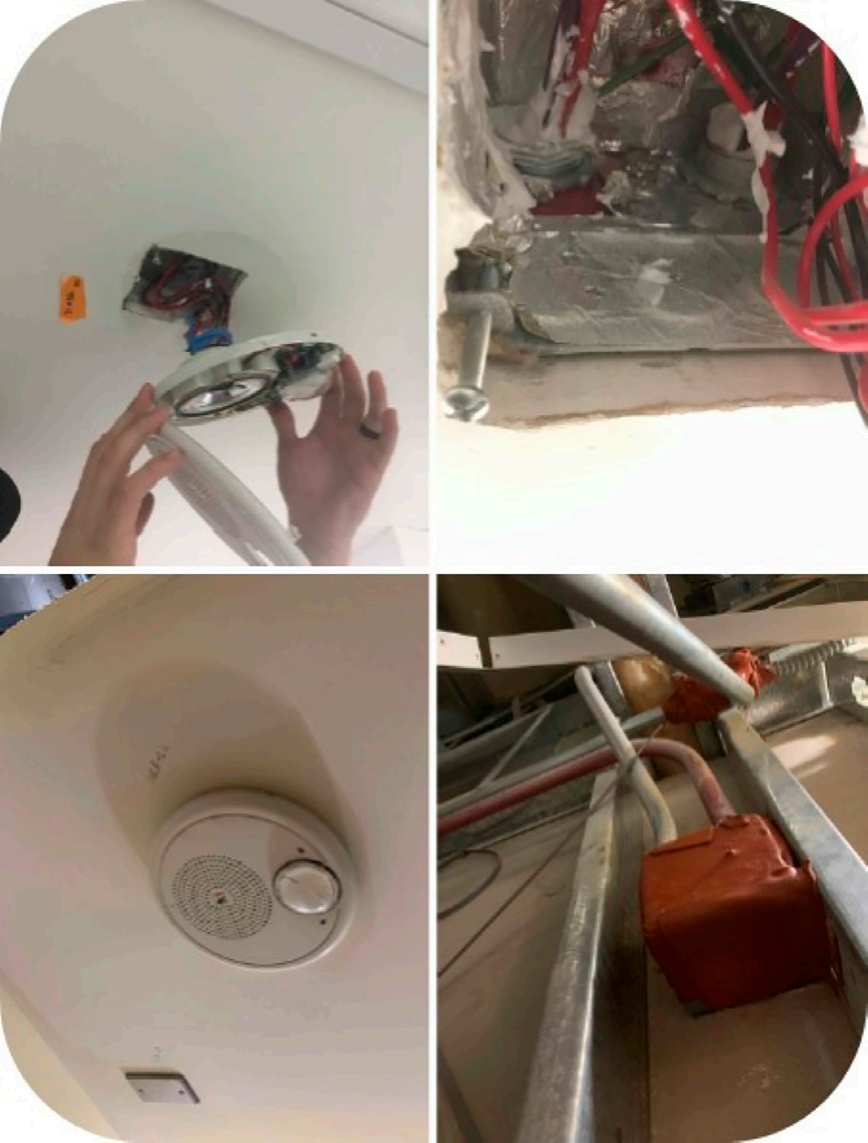
Example of photographs taken to document compliance with sealant requirements.

To achieve commissioning, the team understood that safety standards would need to be verified by various inspection authorities. Knowing that many of the specifications could not be visually verified once construction was complete, the team took comprehensive photographs as evidence that certain physical requirements were satisfied, such as multiple photographs of all sealed penetrations in the walls and ceiling (Figure 2). This visual documentation was stored in construction management software, typical of these projects, along with other functional verification, such as the performance reports from smoke testing all penetrations. This documentation proactively demonstrated the facility fulfilled all requirements. Documenting compliance throughout construction and planning ahead for this critical quality assurance process helps to avoid delays and added difficulties proving that standards are met.

### Project team.

A range of ASU personnel specializing in life science research, safety, and facility management worked closely with architecture, construction, and commissioning personnel contracted by ASU to complete the insectary. Each played a different yet integral role. The team held weekly group meetings for progress updates and design discussions in addition to more targeted supplementary phone calls and E-mails in the intervening days. Intermittent walk-throughs of the space were also scheduled as needed. Over the extended timeline of the project, fulfilling the communication needs of such a large and stratified team proved difficult. It is critical to keep all team members informed of project status, but a flood of meetings and E-mails could hamper progress on the project itself. Additionally, with such a wide range of expertise on the team, conveying technical details to a team member from another trade could be challenging; however, situational awareness of all ongoing activities is critical for coordinated progress. A balance needed to be struck between frequency of communication and level of collaboration among the team.

In any containment facility, early and consistent involvement of the safety team is paramount. Biosafety officers need to assess risk and establish requirements for responsible research with arthropods from the beginning of planning to avoid later complications, delays, or constraints. Safety personnel should be consulted on all decisions, even those without obvious implications for biosafety. Forgoing the input of biosafety officers on the purchase of the rearing chambers at ASU led to the selection of equipment that cannot be controlled at the personnel level. At ACL-3, personnel access restrictions are required for certain pathogens. The purchase of chambers that do not have access control functionality may result in preventing research that is subject to these biosafety regulatory requirements. Although this project demonstrated the importance of consulting safety personnel from early phases, safety personnel learned that they also need to be prepared to offer guidance at every step.

Commissioning should be an active process throughout construction of a containment facility, and delaying this critical quality assurance process could lead to redesign delays and added renovations. At ASU, a commissioning agent was onboarded to the team well into construction and after the team had begun experiencing setbacks, although EHS had advised that they be hired at the onset of the project. Involving an independent, third party with expertise specific to the facility being built and that is focused on the appropriate standards and operational requirements as early as practical helps to avoid major problems late in construction and to ensure the facility will pass all required inspections. Additionally, the presence of this expertise on the team can also relieve that respective burden from internal team members to focus on their intended specialties. A commissioning agent with BSL-3/BSL-4 and insectary-specific experience should be a part of the team from the beginning stages to support design and construction.

The architecture and construction contractors are other pillars of the project team. The importance of their role is clear due to the expertise they can share among the team. Both the design and construction companies selected by ASU had a history of working on life science laboratories, previously with the university and elsewhere, and this experience was key to the success of the project. However, neither had direct experience with a high-containment insectary facility. All members of the project team agreed that this lack of experience led to additional challenges throughout the process. The architecture team solicited a laboratory planner with insectary experience to serve as an internal resource during the design phase but not throughout the project. Reliable access to such expertise could have helped avoid delays for more than a dozen redesigns, including rearranging the floorplan so the laboratory exit did not pass through the containment space and replacing recessed lighting with a sealable alternative, as well as one extended delay when construction was paused for 3 months to reevaluate the plans entirely. Although hiring personnel with this specialized experience may increase labor costs up front, additional unplanned resources will be expended later on to compensate for the gap in expertise. Many public institutions, such as ASU, may be limited by their contract procurement policies to solicit and retain uniquely qualified consultants or specialized companies beyond those frequently used. Despite this barrier, this retrofitting project showed that knowledgeable, in-house experience in designing or building insectaries is key for an uninterrupted process and is worth the initial investment in both project funds and time to clear them through any university processes.

At project outset, the work solicitation for potential contractors should fully and transparently detail the high level of requirements entailed in a specialized facility and the type of expertise needed to deliver on such. Ambiguity may create a divide between the services that contractors plan for and the actual demands of the project. Not only does this ambiguity lead to setbacks and delays, but it places an added burden on the team to adjust to different conditions than anticipated. Companies end up working on the project longer than scheduled and committing more resources than were forecasted; therefore, this complex project demonstrated the importance of setting accurate expectations of exacting project needs.

Each setback caused frustration for the entire team. However, for the researchers and their staff, each setback also further delayed their ability to advance research of public health importance. The date for researchers to move into the insectary was postponed several times, once after the old laboratory space and equipment had been fully packed up in preparation to relocate. Such an unpredictable timeline complicates research projects and laboratory operations as well as securing funding grants. Although challenges are unavoidable, the team learned to keep scientific staff informed and to manage their expectations to mitigate any unintended consequences for the research program. This lesson should also be applied to managing the expectations of anyone who may not be involved in the day-to-day duties but could be affected by the overall progress of the facility.

### Published guidance.

During the design and construction of the insectary, the project team relied on the available guidance documents, as listed in Table 1, to ensure the facility would meet regulations and pass inspection. However, deciphering the necessary requirements across all sources and interpreting how to implement them proved to be an arduous process. The safety team determined that the most effective way to fulfill the requirements was to provide one consolidated, defined list to the rest of the project team. They built the list off of the available guidance and a conservative risk assessment of the worst-case release scenario given their intended research protocols. This requirements list was effective in informing facility design and providing all team members with digestible guidelines specific to the insectary at ASU. Although a range of guidance is available, the team learned that it must be refined into one set of requirements that can be used by the entire team.

Despite all of the requirements presented across the materials, information on how to implement measures to meet these requirements is not readily accessible. Without guidance on how to operationalize the standards, teams are left to essentially plan from scratch. At ASU, this gap led to different interpretations between team members on how best to fulfill various requirements, such as the appropriate light fixtures to qualify as “flush with the ceiling, sealed, and accessed from above.”^9^ The project team ultimately selected surface-mounted lighting to reduce the number of exposed penetrations, but evaluating this decision, and others like it, stalls progress. Moreso, it may be subjective and leave risks unaddressed. The APHIS guidelines specify that metallic, 80-mesh screens, which are stainless steel mesh woven with 80 wires per inch, cover all ventilation ducts to prevent arthropod escape. While contemplating how to install these screens, because specific direction was not provided, the team assessed whether to place them on the inside or outside of the duct covers. The decision was made to install the screens on the outside of the air source to ensure that any escapees could be identified and recaptured. Although placing the screens on the inside would prevent an arthropod from advancing through the ductwork, it would be difficult to recover an escapee caught between the screen and the duct cover, and therefore the risk of a loose arthropod would persist. There was also concern over dust accumulation on the mesh interfering with air flow; therefore, placement on the exterior of the vents allows access for cleaning and routine maintenance. Although comprehensive instructions on how to implement each requirement may not be plausible due to the diversity of arthropod research, implementation guidelines and examples for general risks inherent in biocontainment—such as an arthropod escaping through a gap in the light fixture—are conceivable and valuable for facility design and execution. The standards currently available establish a framework of requirements for an ACL-3 laboratory, and the requirements themselves do not need to be more prescriptive, but additional guidance or published best practices on how to implement and operationalize such requirements would be greatly advantageous.

In search of information on how to satisfy conditions specific to the insectary, the project team leveraged their professional networks. The EHS team in particular reached out to biosafety contacts as well as other similar facilities to harness their experience and seek guidance where the standards left gaps. Open access resources available online were also used when government documents and scientific literature was insufficient.

### Unique solutions.

**Figure 3.**
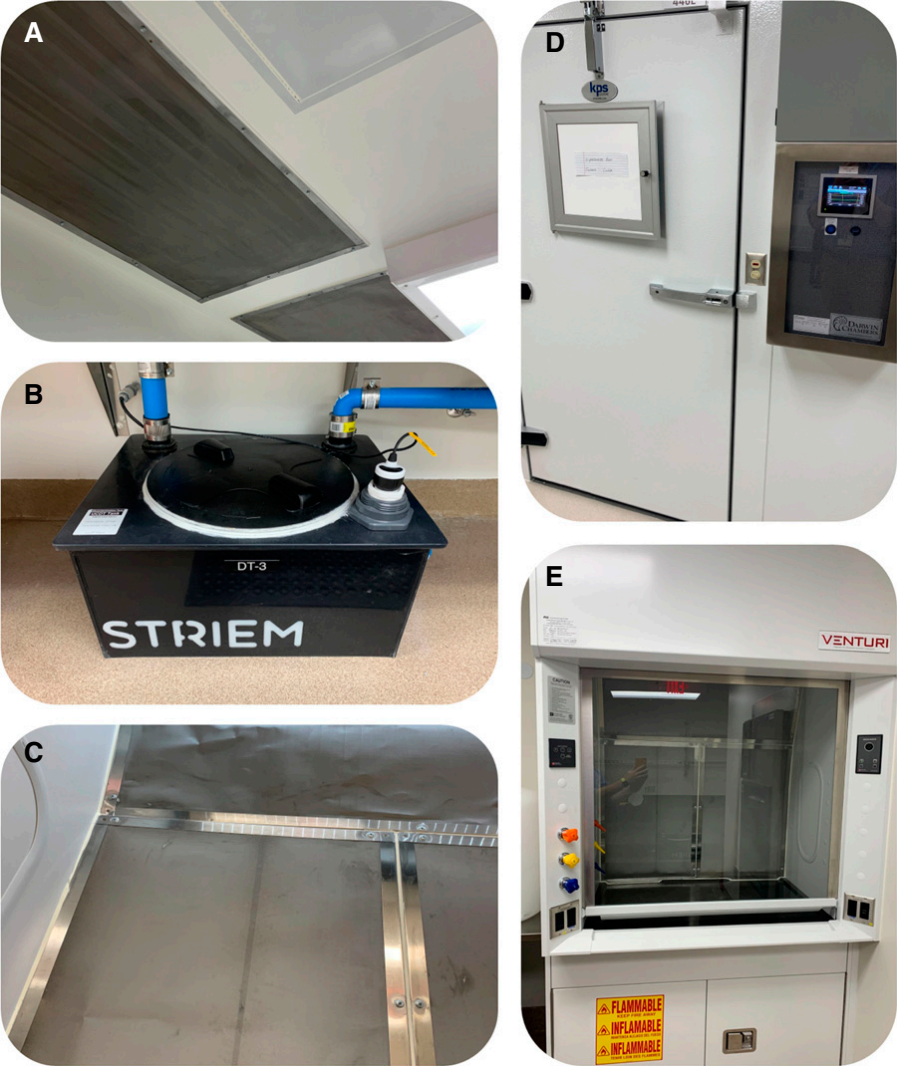
Photographs of custom equipment and specialized designs inside the Arthropod Containment Level 3 insectary at Arizona State University. (**A**) Screens made of 80-mesh installed over ventilation ducts on the side of the containment space and sealable surface mounted light figures. (**B**) Custom kill tanks designed for bleach decontamination of non-infected liquid from arthropod rearing chambers. (**C**) Mesh installed inside body of fume hood to ensure containment in arthropod-handling areas. (**D**) Environmental chambers for rearing arthropods with electronic control panel. (**E**) Laboratory fume hood with body encased in 80-mesh.

After utilizing all of the available guidance, the project team found that there were several requirements that the documentation did not thoroughly address or were unfeasible given their research or facility specifications. To ensure proper containment, the project team innovated new equipment and procedures to mitigate those remaining risks (Figure 3).

The Arthropod Containment Guidelines recommend that “floor drains are modified to prevent accidental release of arthropods and agents,” but they offer no guidance on how this should be accomplished.^9^ Because the APHIS guidelines specify 80-mesh screens to cover ventilation ducts, the project team considered covering the shower drain with the same screen, but concerns arose over the ability for water to properly drain in the event of the use of the shower on a regular basis (the current pathogens in use do not require a shower out of the facility). Nonetheless, the primary objective was to design a facility that would prevent escape to an environmental receptor; therefore, the team proceeded with installing an 80-mesh screen under the shower drain. Separately, the facility emergency shower was located in the main corridor of the space, where there was no floor drain. To address the potential flooding hazard in the event the emergency shower was required, the facility was stocked with enough spill kits to absorb the large volume of water should the emergency shower need to be used and avert damage to the space or compromise worker safety.

Another unaddressed requirement was the need to ensure secondary containment when handling infected arthropods in the vicinity of a fume hood. Through their risk assessment, the team determined that the hood needed an added barrier to prevent a flying arthropod that has escaped primary containment from reaching parts of the hood where it could not be found and captured. They considered adding a screen to the hood sash to prevent any escapees from entering the hood at all but assessed that passing necessary chemicals through a small door in the mesh barrier would create an additional risk to the worker. For this reason, the team devised a mechanism to fit the inner body of the hood with 80-mesh and tested ventilation to ensure there was no interruption in airflow. This added screen layer assures secondary containment and avoids interfering with any other protocols when handling infected arthropods.

Available guidance for ACL-3 laboratories offers “treatment with 10% hypochlorite or by heat, autoclaving, or incineration” as appropriate options for decontamination of any material to be disposed through the sewer. The facility at ASU is not equipped with an effluent decontamination system; nor is their autoclave equipped to handle the large volumes of water expected to be used for rearing mosquitoes. After discussing options with a professional contact at another insectary, the safety team decided to design custom decontamination tanks to address this need. They worked with engineers at a private manufacturer of plumbing products to construct high-density polyethylene tanks with a 21.5-gallon capacity. Non-infected liquid from the rearing chambers is channeled into these tanks and mixed with a bleach solution to provide an innovative solution for decontamination before removal from containment.

In assessing the risk of decontaminating infected material within a biosafety cabinet (BSC), the safety team was unconvinced that a bleach protocol as suggested by the guidance would sufficiently mitigate infection risks. Growing the malaria parasite in blood cultures forms congealed clumps, and so there was concern that the typical bleach solution may not penetrate enough to maintain an acceptable level of efficacy and cannot be autoclaved because of the release of toxic fumes.^10^ While operating at the BSL-2 level, the team devised a protocol to continue decontaminating all blood culture waste with bleach but treat it as chemical waste and decontaminate the containment vessel at the facility exit. When the facility is used at the ACL-3 level in the future, the team plans to validate a new decontamination procedure using an antiseptic iodine solution within the BSC followed by steam sterilization.

## CONCLUSION

Retrofitting a high-containment insectary into a previously uncommissioned laboratory presented a myriad of challenges for the project team at ASU. Countless operational and engineering solutions had to be devised along the way to compensate for deficiencies in the space, vague guidelines, and a stratified team. Although completion of the insectary was delayed, the team thoroughly assessed potential risks and ultimately delivered a facility that enables researchers at ASU to safely perform critical arthropod research of public health importance. By sharing the lessons learned by an experienced team, we hope to bolster the biosafety of forthcoming insectaries and to offer helpful guidance to the teams undertaking their construction.
